# Safety and Tolerability of *Withania somnifera* Root Extract in Healthy Male Participants: A Pilot Randomized, Double‐Blind, Placebo‐Controlled Clinical Trial

**DOI:** 10.1002/fsn3.71388

**Published:** 2025-12-26

**Authors:** Narendra Vaidya, Ramshyam Agarwal, Pankaj Kshirsagar, Gayatri Ganu, Dheeraj Nagore, Anirudh Mehta, Ashit Vora, Sujit Nair

**Affiliations:** ^1^ Lokmanya Medical Research Center and Hospital Pune India; ^2^ MPREX Healthcare Pvt. Ltd. Pune India; ^3^ Phytoveda Pvt. Ltd. Mumbai India; ^4^ Viridis Biopharma Pvt. Ltd. Mumbai India

**Keywords:** ashwagandha, clinical trial, nutraceutical, safety, tolerability, *Withania somnifera*

## Abstract

*Withania somnifera* (WS), also known as Ashwagandha in Ayurveda, is valued for its anti‐inflammatory, antioxidant, adaptogenic, and memory‐enhancing properties. This study assessed the safety of standardized Ashwagandha root extract in healthy adult participants over 180 days. A randomized, placebo‐controlled, double‐blind trial was conducted on 40 healthy adult participants (50–70 years), randomized 1:1 to receive 200 mg WS extract or placebo capsules twice daily for 180 days. Each WS capsule contained ≥ 5.00 mg of withanolides, standardized by HPLC‐PDA per USP‐NF monograph. Safety outcomes were assessed at screening and day 180. Hematological, hepatic, renal, and lipid profiles remained within normal ranges with no clinically significant changes compared to screening. Testosterone levels significantly increased (15.7%) in male participants in the WS group. Thyroid hormone levels, inflammatory marker (CRP), and NT‐pro BNP levels showed no adverse changes. CRP decreased by 51.1%, and NT‐pro BNP by 28.70% in the WS. Slight improvement in immunological markers (CD3, CD4, CD8) was also noted. The vital signs remained stable, with no clinical abnormalities detected on chest X‐ray or ECG. Anthropometric parameters were not clinically changed after treatment. Taken together, the study concluded that WS root extract capsules (200 mg twice daily for 6 months) were well‐tolerated and safe, with no adverse effects reported. This double‐blind, placebo‐controlled study with extended follow‐up provides a comprehensive evaluation of the long‐term safety profile of WS supplementation.

**Trial Registration:** Clinical Trial Registry‐India (CTRI) approval number: CTRI/2023/11/059395

## Graphical abstract

*Withania somnifera* (WS) or Ashwagandha is valued for its anti‐inflammatory, antioxidant, adaptogenic, and memory‐enhancing properties. This randomized, placebo‐controlled and double‐blind study assessed the safety of standardized WS root extract in healthy adult participants over 180 days. The study concluded that WS root extract (200 mg capsules twice daily for 6 months) was well‐tolerated and safe, with no adverse effects reported.

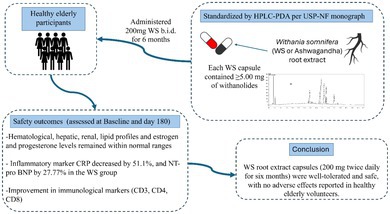

## 1 Introduction

*Withania somnifera* (L.) Dunal, belonging to the Solanaceae Family, also known as the “Indian Ginseng”, “Winter cherry”, “Ashgandh” or “Ashwagandha” is a widely used herb, renowned globally for its adaptogenic and immunomodulatory health benefits (Paul et al. 2021). It is distributed in India, Sri Lanka, the Middle East, China, Africa, and the warm areas of Europe and Australia (Saleem et al. 2020). The trust reposed in the beneficial properties of this herb is based on ancient Indian traditional systems of medicine, including Ayurveda, Unani and Siddha, which have a variety of medicinal effects attributed to their different uses (Joshi and Joshi 2021). WS consists of several bio‐actives, including alkaloids, saponins, tannins, withanolide glycosides (sitoindosides), and withanolide aglycones (Misico et al. 2011; Shinde et al. 2023; Girme et al. 2020). More than 12 alkaloids, 40 withanolides such as withanolide A, B, Withaferin A, 12‐Deoxy‐withastramonolide, etc., and several sitoindosides have been isolated from roots, aerial parts and berries of different species of *Withania* (Modi et al. 2022). Reports of WS date back to 1000–1500 B.C. in “Ayurveda” and it is used as a rejuvenator, tonic, weight promoter, aphrodisiac, for eyes, abdomen and skin diseases, insomnia, gout, wounds, etc. (Meher et al. 2016). In the “Siddha” medicinal system, WS is claimed as an anti‐inflammatory, hypnotic, diuretic and tonic, whereas in “Unani” medical literature, the actions of “Asgand” are bulk promoting, nervine tonic, and hypnotic (Berra et al. 2024). Also, in “Homeopathy”, WS is used in debility, fatigue, and spermatorrhea (Joshi and Joshi 2013). Clinical and preclinical trials on the roots and extract of WS reveal a multitude of biological activities such as anti‐cancer, adaptogenic, anti‐stress, immunomodulatory, anti‐bacterial, anti‐aging, cardioprotective, neuroprotective, etc. (Mahdi et al. 2011; Deshpande et al. 2020; Baker et al. 2022; Malik et al. 2009; Mohanty et al. 2004; Choudhary et al. 2015; Saha et al. 2024; Pandit et al. 2024).

The mechanism of WS in diseases such as chronic inflammation in aged individuals (inflammaging) and neurodegenerative disorders has been elucidated previously, which highlights the role of WS in alleviating these disorders at transcriptomic and proteomic levels (Saha et al. 2024; Basudkar et al. 2024). Effects of WS as an adaptogenic herb in mitigating stress and inflammaging have also been elucidated in recent years (Basudkar et al. 2024; Machín et al. 2023).

The global WS market is set to grow from $433 M in 2022 to $1187 M by 2032 (CAGR 10.9%). In the United States, WS was the third top‐selling natural product in retail, with $13.7 M in sales, after elderberry and cannabidiol (Dadge et al. 2023). WS demand is rising with insomnia cases. The US National Center for Complementary and Alternative Medicine (NCCAM) prioritizes WS for research, and it is listed in major pharmacopeias (Wijeratne et al. 2014). However, some European regulators, including Denmark's Danish Veterinary and Food Administration (DVFA), have raised safety concerns, banning WS over potential adverse effects (Patwardhan et al. 2024). Therefore, assessing the safety and/or tolerability of WS root extract is crucial to understanding health‐beneficial effects, or otherwise, in healthy individuals worldwide.

Several clinical studies were performed concerning the safety of WS root extract on healthy and diseased participants. Raut et al. (2012) performed an open‐label study on 18 participants to assess the safety of a WS formulation with escalating doses over 30 days. WS was deemed safe based on hematological and biochemical assessments and showed lipid‐lowering and muscle‐strengthening effects (Raut et al. 2012). In a separate study, Vaidya et al. (2024) found that 1000 mg/day of WS root extract (7.5 mg withanolides/capsule) was safe for male volunteers over 30 days. Verma et al. (2021) conducted a double‐blind, placebo‐controlled, parallel‐group safety study of WS at 300 mg twice a day for 2 months. The study's results did not indicate any toxic effect in 80 healthy (40 male and 40 female) volunteers (Verma et al. 2021). Gopukumar et al. (2021) reported the effect of a WS root extract capsule of 300 mg once a day for 90 days in 130 healthy subjects and no AEs were observed during this study. Langade et al. (2019) reported a 10‐week placebo‐controlled, double‐blind trial to assess the safety and effectiveness of 300 mg WS root extract, administered twice daily, in 60 patients experiencing insomnia. The authors revealed that WS root extract is well‐tolerated in patients without adverse effects (Langade et al. 2019). Biswal et al. (2013) investigated the effects of WS (6 g daily in divided doses) on cancer patients undergoing conventional therapies and adverse effects of oral intolerance, gastritis, and flatulence were reported. In 2023, AYUSH, Government of India, published a Safety Dossier on WS, summarizing findings from various studies on its potential adverse effects. After reviewing 27 toxicity studies, the dossier concluded that WS root powder (extract) is safe for human consumption at doses of up to 2000 mg/kg body weight, with no observed adverse effects (Ruknuddin et al. 2023 ). Similarly, in 2024, AYUSH published a revised version of the Safety Dossier in which they have further consolidated all the relevant studies that have been published with respect to the safety aspect of WS (Ministry of Ayush, Government of India, New Delhi 2024). The Food Safety and Standards Authority of India (FSSAI) regulates nutritional supplements and food products in India. In Schedule II, FSSAI recommends 3–6 g of WS root powder or 0.5–1 g of extract daily for adults. Systematic reviews and meta‐analyses confirm WS is safe with no serious adverse events reported (Bonilla et al. 2021).

In Ayurveda, WS leaves are recommended primarily for external use. Ethnopharmacological records indicate that a leaf paste can be used for application to painful areas, and boiled leaves are sometimes used topically to relieve pain and treat boils (Indian Ministry of AYUSH. Government of India 2021). However, traditional Ayurvedic texts advise against the oral use of WS leaves due to potential toxicity concerns. Despite this, some commercial products mix aerial parts, such as leaves or stems with root material, which may contribute to adverse effects. Ayurveda recommends that only roots be used for oral administration and therapeutic purposes. Recently, the Indian Ministry of AYUSH issued an advisory recommending against the use of WS leaves, citing insufficient evidence to support their medicinal use (Vaidya et al. 2024; Bonilla et al. 2021).

Although multiple clinical trials were performed on WS extract for various biological activities, the long‐term safety of USP‐NF standardized WS root‐only extract is still missing. Further, the literature also lacks the effect of standardized WS root extract on the male/female reproductive system, thyroid function, and immune system. In the current research work, the safety of HPLC‐PDA standardized WS root extract was evaluated on healthy adult participants based on a randomized, placebo‐controlled, double‐blind clinical study.

## 2 Material and Methods

### 2.1 Study Design and Objectives

**FIGURE 1:**
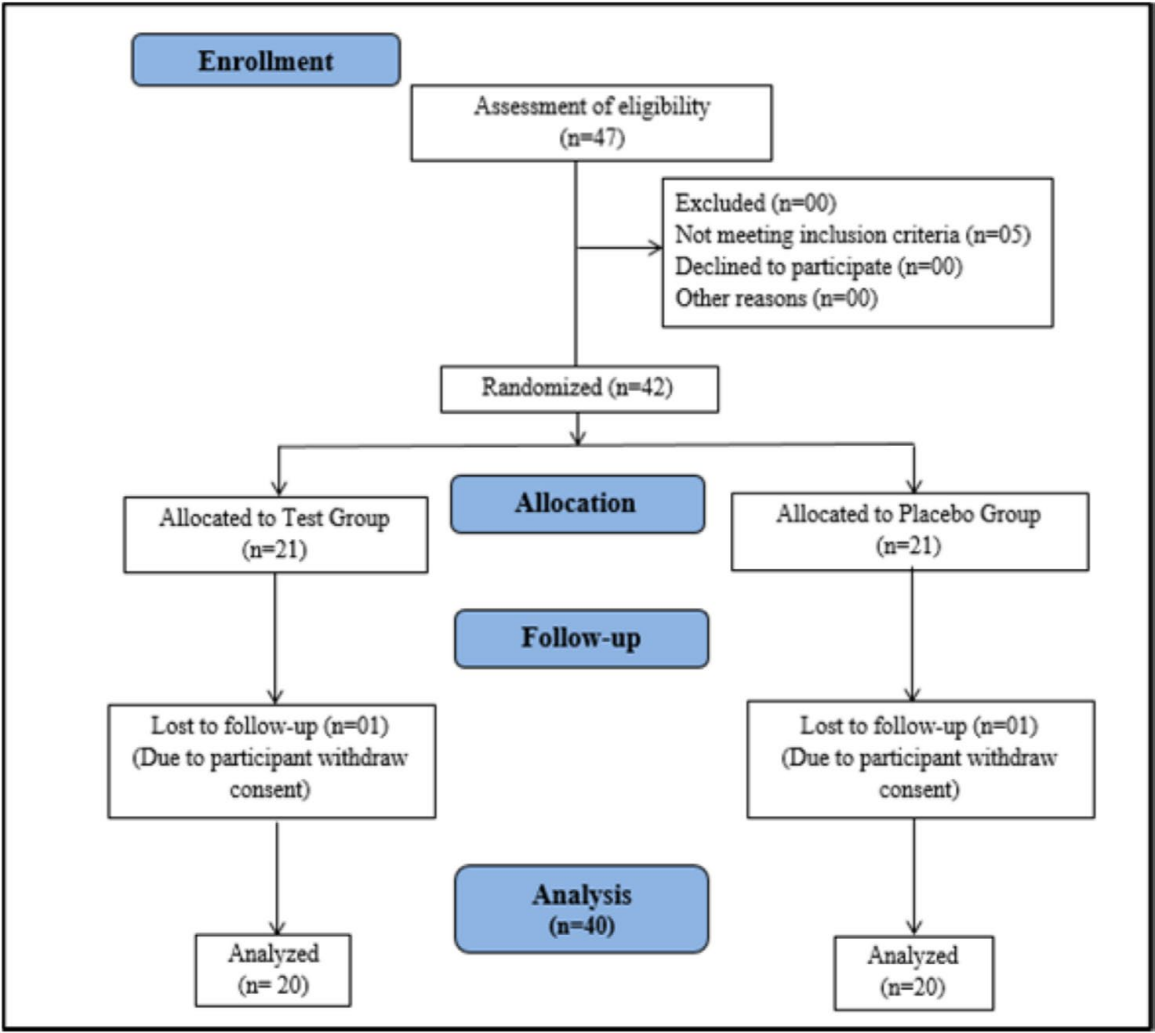
Consolidated Standards of Reporting Trials (CONSORT) diagram for the study.

A randomized, parallel‐group, double‐blind, comparative, placebo‐controlled clinical trial was performed to assess the safety of WS root extract (LongeFera) capsules on healthy adult human volunteers. The study's primary objective was to investigate the safety of WS root extract capsules when administered to adult male and female participants. The secondary objective was establishing safety and tolerability by recording any AEs. In this study, 42 participants were randomly assigned into the treatment (WS root extract capsules) and the placebo groups, with 21 participants per group. However, 40 participants completed the study (20 in each group), including 36 males and 4 females. The study duration was 180 days, and the safety of the investigational product was compared between both groups (Figure 1).

### 2.2 Ethics Approval and Registration of Clinical Trial

Institutional Ethics Committees (IECs) of Lokmanya Medical Research Centre Pune‐411,033, Maharashtra, India, provided approval for the study (clinical trial ethics approval protocol no. MHC/CT/23‐24/025, approved on 25/10/2023; Ethics committee Registration No. ECR/175/Inst/MH/2013/RR‐19). The Drug and Cosmetic Act of 1940, the Drug and Cosmetic Rules of 1945, the World Medical Association's (WMA) Declaration of Helsinki, the International Conference on Harmonization's (ICH) guideline on Good Clinical Practice, and other ethical principles and guidelines were followed during the conduct of the clinical trial (ICH‐GCP). The investigational product was manufactured in a GMP (Good Manufacturing Practice)‐approved facility. The clinical trial and protocol were registered with the Clinical Trial Registry‐India (CTRI) with approval number CTRI/2023/11/059395 (Registered on: 01/11/2023). The approved clinical trial protocol was not altered during the study period.

### 2.3 Informed Consent

Participants received an explanation of the clinical trial design, expected benefits, and potential hazards of the study before participating. The participants could understand the format and language of this information. Participants were duly informed that they could leave at any time during the study. Each participant gave written consent before they were enrolled in this study. Participants were informed about the study's objectives and methods, possible risks and benefits, confidentiality policies, and the institutional ethics committees' contact details and study investigators' contact details. All personal data and study information were tagged as well as securely archived to maintain confidentiality of participants. The anonymity of the participants was preserved in all publications and study reports. The data were only accessible to approved study personnel.

### 2.4 Study Participants, Inclusion, and Exclusion Criteria

Forty‐two participants were recruited in a double‐blind, placebo‐controlled, randomized clinical trial of WS root extract capsule to assess long‐term safety upon oral consumption. In this study, healthy adult men and women ages 50–70 years with a BMI < 30.00 kg/m^2^ were screened and included. During screening and prior to administration of the WS capsules, each volunteer underwent baseline tests that included an X‐ray chest (PA view), ECG (Electrocardiogram), complete blood count (CBC), liver function test (LFT) and kidney function test (KFT), clinical biochemistry, and urine analysis. During the clinical trial period, the well‐being and safety of each healthy participant was monitored, and AEs were recorded. Participants meeting the following inclusion criteria were enrolled: (a) Males and females between 50 and 70 years of age (both inclusive); (b) Participants were included with or without comorbidity (type 2 diabetes and hypertension); if comorbidity was present, it was either one or both of the following: In the case of type 2 diabetes, the hemoglobin A1c (HbA1c) level was ≤ 8% on a stable regimen (i.e., same drug and same dose) for at least 12 weeks before the screening visit. In the case of hypertension, systolic BP was between 120 and 160 mmHg (both inclusive), and diastolic BP was between 70 to 100 mmHg (both inclusive) while on a stable regimen (i.e., same drug and same dose) for at least 12 weeks before the screening visit. (c) Participants who had committed to showing up for follow‐up appointments regularly. (d) Participants who could read and write in English.

The study's exclusion criteria were as follows: (a) Individuals with a known record of hypersensitivity to herbal extracts or dietary supplements were excluded from the study; (b) women who were nursing or pregnant, those who were not using sufficient forms of birth control, and those who tested positive for urine pregnancy test were also not included; (c) participants with a history or presence of malignancy within the last 2 years were excluded; (d) Participants with asthma, uncontrolled diabetes mellitus, or hypertension were not included in the study; (e) Participants who were on immunosuppressive drugs (e.g., corticosteroids) and/or antihistamines (e.g., antiallergics) during the last 7 days before the commencement of the study were excluded; (f) Participants with any history or immediate family history of schizophrenia, other psychotic or neurological illness, severe personality disorder, or other significant psychiatric disorder that the investigator believed could impact the ability of the participant to comply with requirements of the study; (g) Participants with deranged biochemical parameters such as AST (SGOT), ALT (SGPT) > 1.5× ULN (Upper Limit of Normal); Total bilirubin > 1.5 mg/dL; Creatinine > 2 mg/dL; Alkaline phosphatase > 5× ULN were eliminated from the study; (h) Participants with known or suspected history of a diagnosed dependence disorder, heavy alcohol consumption, use of an illicit drug, or non‐prescribed use of any prescription drug were not included; (i) Participants who were currently using WS in any form or any other herbal nutraceutical were also excluded from this study.

### 2.5 Clinical Study Procedure

The participants' demographic and medical history information, such as age, sex, height and body weight were recorded during the screening of participants. Each participant was subjected to a body examination, vital signs, clinical examination, anthropometric evaluation, hematological and biochemical evaluation, ECG, and chest X‐ray (P/A radial). Each participant filled out the case report form (CRF) to report medication interaction and concurrent medical conditions. If participants met all inclusion/exclusion criteria, they were then enrolled in the clinical trial with informed consent. Two groups of 20 participants each were randomly assigned to receive either a WS root extract capsule (200 mg) or a placebo capsule in a 1:1 ratio (20 people in each group) (Figure 1). The treatment duration was 180 days, and the product's safety was compared with screening/180‐day data and between both groups. Patients were instructed to consume the designated capsule twice daily for 30 min after breakfast and dinner for 180 days. The dose of WS root extract capsule was estimated based on data from preclinical in vivo studies for safety and efficacy and clinical pharmacokinetic study of WS root extract capsule in male and female participants at 400 mg dose (Sharma et al. 2025). Ayurveda, homeopathy, Siddha, Unani, and nutraceutical supplements were not permitted during the clinical study period. None of the study participants had concomitant diseases; hence, no concomitant treatment was required.

The primary safety assessment was carried out by assessment of any changes in the clinical laboratory examination of complete blood count, renal function test, liver function test, thyroid profile, X‐ray (Chest), ECG, and vital signs at screening and the end of 180 days. Further, additional blood analyses such as glycaemic status, immune system biomarkers, CRP level, cortisol, and male reproductive system hormone (testosterone) were also performed at screening and end of the study from participants of both the groups. A trained phlebotomist collected blood samples during the screening and day 180 visits, with each sample having a maximum volume of 10 mL. The blood was collected and divided into 5 mL in an EDTA tube and 5 mL in a plain tube. A centralized National Accreditation Board for Testing and Calibration Laboratories (NABL) accredited laboratory (My Labs Healthcare, Office No 15, 1st Floor, Ganesham Commercial, Sai Nagar Park, Pimple Saudagar, Pimpri‐Chinchwad, Pune‐411027, Maharashtra) processed the safety assessment of samples. The investigators strictly adhere to the regulatory authority and IRB‐approved protocol. The study monitor reported all protocol “violations” (for inclusion/exclusion) and protocol “deviations” (related to study procedures), and a protocol deviation/violation log was filled for every such case. The safety and tolerability of the investigational product were assessed by reporting and analyzing the AEs that occurred, vital signs, etc.

### 2.6 Adverse Events (AE)

All adverse events‐related signs or symptoms were recorded in the AE form as part of the CRF. In particular, the information included details of the administration of the investigational product and details of AE with its date, onset time, causality, frequency, severity, outcome, and if any treatment or diagnostic steps were taken. All AEs were followed until resolved completely.

### 2.7 Investigational Product

#### 2.7.1 Quality Control of WS Extract (LongeFera)

Roots of WS were received from Madhya Pradesh, India, and validated by a certified taxonomist at Botanical Survey of India, Jodhpur, India. A voucher specimen was deposited (BSI/AZRC/I.12012/Tech/19‐20/PI.Id/671). The root material was washed and pulverized, and a rigorous investigation was conducted to determine the content of total withanolides, contaminants, and heavy metals. The powder was thereafter extracted using ethanol: water (8:2 v/v) at 60°C; following which the extract was processed to obtain a powder. The extract was analyzed for withanosides and withanolides content using HPLC‐PDA as per the USP‐NF “*WS* root extract monograph”. The USP‐NF standardized root extract of WS containing not less than 2.50% w/w total withanolides, which is globally marketed by Phytoveda USA and Phytoveda Pvt. Ltd., India, with the brand name LongeFera, was used for manufacturing of Investigational Product.

#### 2.7.2 Manufacturing of Investigational Product (WS Root Extract Capsules)

The WS extract was formulated in veggie capsules (Size “00”) with a dosage of 200 mg and manufactured in a state‐of‐the‐art GMP‐certified facility. The placebo capsule contained an equal amount of Maltodextrin. The capsules were filled in a child‐lock container and used as an investigational product in randomized clinical trials.

### 2.8 Statistical Considerations and Analysis

#### 2.8.1 Sample Size Consideration

Sample size estimation was on the basis of available literature evidence. The therapeutic dose was considered for dosage selection considering the long‐term safety assessment approach for deciding study duration.

#### 2.8.2 Demographic and Baseline Information

The analysis was conducted using a Student's *t*‐test. The continuous variable, age, was comprehensively summarized using descriptive statistics, including the number of observations, mean, and standard deviation, with a 95% confidence interval assuming a normal distribution. Gender was assessed separately using the Chi‐squared test. All safety parameters were checked for normality using the Kolmogorov–Smirnov test. Data on weight, BMI, waist circumference, hip circumference, vital signs, and all laboratory parameters were analyzed using a dependent Student's *t*‐test. The statistical analyses of X‐ray and ECG were performed using the Chi‐squared test. The AEs were denoted as the number and frequency of events in a study group.

#### 2.8.3 Statistical Analysis

All study analyses were carried out using SPSS Version 10.0. The data were summarized with descriptive statistics (number of patients, mean, standard deviation, minimum, median, and maximum) for continuous endpoints and frequency and percentage for categorical endpoints. The safety population consisted of all patients enrolled in the study who received at least one dose of study products, that is, the mITT population. AEs and SEs were summarized by counting both the number of separate events and the number of patients experiencing events during the study period. Furthermore, similar summaries were stratified according to the seriousness, severity, and relationship to the study medication. The percentage of cases with an event and tolerability was analyzed and compared by the Chi‐squared test.

## 3 Results

### 3.1 Demographic Characteristics and Anthropometric Parameters of Participants

The demographic and anthropometric data of the study population comprised 40 participants. The demographic characteristics, including age, weight, height, BMI, hip circumference and waist circumference, are depicted in Table 1. Each group comprised 20 participants, with the test group including 19 males and 1 female, and the placebo group comprising 17 males and 3 females, all of whom completed the study. The mean age of participants in the WS group was 55.25 ± 5.24 years, whereas in the placebo group, it was 52.25 ± 1.97 years. Screening and Day 180 data suggest statistically insignificant (*p* > 0.05) differences in weight and BMI. However, there was a significant difference in hip and waist circumference observed on Day 180 (*p* < 0.05).

**TABLE 1:** Demographic and anthropometric characteristics of participants.

Demographic details	Screening			Day 180		
	Test	Placebo	*p*	Test	Placebo	*p*
No of participants	20	20	—	20	20	—
Male	19	17	—	19	17	—
Female	01	03	—	01	03	—
Age (Mean ± SD)	55.25 ± 5.24	52.25 ± 1.97	0.022	—	—	—
Weight (kg)	66.69 ± 11.48	65.58 ± 8.28	0.727	66.17 ± 11.19	65.25 ± 7.70	0.754
BMI	24.42 ± 3.78	25.28 ± 2.49	0.397	24.39 ± 3.64	25.23 ± 2.45	0.901
Waist circumference	92.53 ± 12.84	89.95 ± 8.70	0.462	92.33 ± 13.00	91.14 ± 8.78	< 0.001
Hip circumference	94.65 ± 13.54	95.95 ± 10.90	0.740	93.75 ± 13.45	96.80 ± 11.01	< 0.001

*Note:* Data were analyzed by the dependent Student's *t*‐test. Significant at *p*‐value < 0.05.

### 3.2 Effect of WS on Hematological Parameters

A hematological analysis was conducted at screening and day 180 for all participants. After 180 days, both groups demonstrated statistically significant changes, with increased RBC counts and decreased ESR compared to screening. The test group also showed a statistically significant increase in total leukocyte count. However, these changes were not clinically significant. All other hematological parameters were both statistically and clinically non‐significant (*p* > 0.05). All parameters including RBC, ESR, and TLC remained within reference ranges throughout the study. These findings support a favorable safety profile for both the test and control groups. Hematological data are presented in Table 2.

**TABLE 2:** Assessment of hematological parameters during screening and upon completion of treatment at day 180 in healthy adult participants.

Parameters	Test (*n* = 20)		Placebo (*n* = 20)		Reference range
	Screening	Day 180	Screening	Day 180	
**Complete blood count**					
Total leukocyte count (cell/mm^3^)	6890.00 ± 1442.89	7877.25 ± 1984.78^a^, ^b^	6632.50 ± 2123.40	6200.00 ± 1252.14	4000–11,000
Neutrophils (%)	67.10 ± 6.32	66.15 ± 8.57	62.75 ± 9.66	60.70 ± 7.69	40–75
Lymphocytes (%)	25.55 ± 4.84	25.85 ± 7.75	29.50 ± 9.08	30.80 ± 8.21	20–40
Monocytes (%)	4.65 ± 1.76	5.05 ± 1.61	4.35 ± 1.50	5.15 ± 1.57	2–10
Eosinophil (%)	2.70 ± 0.86	2.95 ± 1.00	2.90 ± 1.07	3.35 ± 1.09	1–6
Basophils (%)	0.00 ± 0.00	0.00 ± 0.00	0.00 ± 0.00	0.00 ± 0.00	0–1
Total RBC count (m/mm^3^)	4.70 ± 0.66	5.05 ± 0.56^a^	4.53 ± 0.71	4.82 ± 0.32^a^	4.7–6.0
Platelets (10^3^/μL)	234.40 ± 72.98	227.25 ± 72.52	278.90 ± 66.48	252.60 ± 51.95	150–450
Hemoglobin (gm/dL)	13.32 ± 1.81	13.87 ± 1.49	12.52 ± 1.40	12.99 ± 1.41	13.2–16.6
Hematocrit (%)	41.13 ± 4.94	42.83 ± 4.26	38.99 ± 5.17	40.05 ± 4.27	42–52
ESR (mm/h)	14.30 ± 3.40	11.15 ± 3.98^a^	13.15 ± 2.46	9.65 ± 2.46^a^	0–20

*Note:* Data is represented as Mean ± SD (percent change). The data was analyzed within a group using the dependent Student's *t*‐test and Wilcoxon signed‐rank test and between groups using the independent Student's *t*‐test and Mann–Whitney *U* test. Statistically significant at *p*‐value < 0.05.

^a^ Significant *p*‐value within the group.

^b^ Significant *p*‐value between groups.

### 3.3 Effect of WS on Hepatic and Renal Function

Liver and renal function tests between the test and control groups over the 180 days demonstrated that all the values stayed within the normal physiological range in both groups, and these changes were not clinically significant, indicating a favorable safety profile for both the test and control group (Table 3).

**TABLE 3:** Liver and renal function test upon treatment with WS root extract capsule and placebo at screening and day 180.

	Test (*n* = 20)		Placebo (*n* = 20)		Reference range
	Screening	Day 180	Screening	Day 180	
**Liver function test**					
Total bilirubin (mg/dL)	0.67 ± 0.22	0.73 ± 0.36	0.64 ± 0.36	0.60 ± 0.27	0.0–1.1
Direct bilirubin (mg/dL)	0.15 ± 0.05	0.18 ± 0.07	0.17 ± 0.11	0.16 ± 0.08	NMT 0.4
Indirect bilirubin (mg/dL)	0.53 ± 0.18	0.55 ± 0.30	0.49 ± 0.30	0.44 ± 0.21	0.1–1.2
SGOT/AST (U/L)	31.31 ± 11.41	33.33 ± 9.62	32.85 ± 15.75	38.23 ± 20.59	0–49
SGPT/ALT (U/L)	28.58 ± 15.87	32.44 ± 20.62	28.58 ± 16.00	30.01 ± 15.05	0–49
Alkaline phosphatase (ALP) (U/L)	208.54 ± 59.06	175.12 ± 45.95^a^	193.32 ± 52.10	161.07 ± 36.12^a^	63–306
Total protein (g/dL)	7.14 ± 0.60	7.43 ± 0.68^a^	7.18 ± 0.71	7.43 ± 0.46	6.0–8.3
Albumin (g/dL)	4.18 ± 0.17	4.31 ± 0.19^a^	4.21 ± 0.28	4.37 ± 0.23^a^	3.5–5.5
Globulin (g/dL)	2.95 ± 0.50	3.12 ± 0.60	2.97 ± 0.58	3.06 ± 0.37	1.8–3.6
A/G ratio	1.45 ± 0.22	1.43 ± 0.28	1.47 ± 0.30	1.45 ± 0.19	—
**Renal function test**					
Blood urea nitrogen (mg/dL)	10.88 ± 2.69	12.61 ± 2.97^a^	9.89 ± 3.45	10.20 ± 2.72	7–21
Blood urea (mg/dL)	23.29 ± 5.76	27.00 ± 6.35^a^	21.19 ± 7.39	21.83 ± 5.83	10–50
Serum creatinine (mg/dL)	0.89 ± 0.13	0.96 ± 0.15^a^	0.87 ± 0.17	0.93 ± 0.15	0.7–1.4
Uric acid (mg/dL)	5.69 ± 1.28	5.69 ± 1.26	5.53 ± 1.20	5.39 ± 0.99	3.0–7.2

*Note:* Data is represented as Mean ± SD (percent change). The data was analyzed within the group using the dependent Student's *t*‐test and between groups using the independent Student's *t*‐test. Statistically significant at *p*‐value < 0.05.

^a^ Significant *p*‐value within the group.

### 3.4 Effect of WS Root Extract on Lipid Profile

The lipid profile between the test and placebo groups over 180 days demonstrated that all the values remained within the normal physiological range in both groups. Total Cholesterol and LDL significantly increased in the placebo group; the changes were non‐significant in the test group, indicating that the WS root extract does not have any significant effect on lipid levels upon repeated dose oral consumption for up to 6 months (Table 4).

**TABLE 4:** Lipid profile at screening and day 180 upon oral administration of WS root extract and placebo capsule in healthy adult participants.

Lipid profile					
Parameters	Test (*n* = 20)		Placebo (*n* = 20)		Reference range
	Screening	Day 180	Screening	Day 180	
Total Cholesterol (mg/dL)	171.01 ± 36.26	177.37 ± 42.59	167.00 ± 40.06	182.55 ± 33.07^a^	Desirable level | < 200
Triglycerides (mg/dL)	166.39 ± 79.87	126.71 ± 33.30	154.47 ± 77.34	155.39 ± 77.15	Normal: < 165
High‐density lipoprotein (HDL) (mg/dL)	49.94 ± 9.65	52.51 ± 11.54	46.53 ± 8.37	46.70 ± 5.62	Normal: 35–80
Low‐density lipoprotein (LDL) (mg/dL)	87.79 ± 35.23	92.33 ± 37.67	89.58 ± 30.12	104.77 ± 31.99^a^	Optimal < 100 Near/Above Optimal 100–129
Very Low‐Density Lipoprotein (VLDL) (mg/dL)	33.28 ± 15.97	25.21 ± 6.66	30.89 ± 15.47	31.08 ± 15.43	6–38

*Note:* Data is represented as Mean ± SD (percent change). The data was analyzed within a group using the dependent Student's *t*‐test and between groups using the independent Student's *t*‐test. Statistically significant at *p*‐value < 0.05.

^a^ Significant *p*‐value within the group.

### 3.5 Effect of WS Root Extract Capsule on the Male Reproductive Hormone

Testosterone levels were evaluated in study participants at screening and upon treatment with WS on Day 180. There was a significant improvement (*p* < 0.05) in testosterone levels in males in the test group than in the placebo group (Table 5). This indicates that WS root extract increases the testosterone level and could increase muscle mass. Since the number of female participants was very small (*n* = 1 in the test group and *n* = 3 in the placebo group), no analysis data has been included for female participants.

**TABLE 5:** Male sex hormone (testosterone) at screening and upon treatment with WS root extract capsule in participants.

Parameters	Test (*n* = 19)		Placebo (*n* = 17)	
	Screening	Day 180	Screening	Day 180
Testosterone (ng/dL)	471.33 ± 218.50	555.12 ± 270.72^a^, ^b^	489.33 ± 306.10	434.77 ± 249.85
%Change	17.78% (Improvement)		11.15% (Reduction)	

*Note:* Data is represented as Mean ± SD (percent change). Data were analyzed by the dependent Student's *t*‐test and Wilcoxon signed‐rank test for within groups and independent Student's *t*‐test and Mann–Whitney *U* test for between groups. Statistically significant at *p*‐value < 0.05.

^a^ Significant *p*‐value within the group.

^b^ Significant *p*‐value between groups.

### 3.6 Effect of WS Root Extract on Thyroid Hormones

Thyroid function tests between the test and control groups over the 180 days demonstrated that all the values stayed within the normal physiological range in both groups and demonstrated statistically significant changes, with decreased Triiodothyronine and Thyroxine compared to screening. However, these changes were not clinically significant. All other thyroid parameters were both statistically and clinically non‐significant (*p* > 0.05), indicating that the WS root extract was safe for thyroid gland function upon repeated oral dose exposure for 6 months (Table 6).

**TABLE 6:** Thyroid function test upon oral administration of WS root extract and placebo capsule in healthy adult participants.

Thyroid profile					
Parameters	Test (*n* = 20)		Placebo (*n* = 20)		Reference range
	Screening	Day 180	Screening	Day 180	
Triiodothyronine (T3) (ng/dL)	160.20 ± 22.82	142.86 ± 23.26^a^	158.65 ± 33.01	141.91 ± 29.09	60–215
Thyroxine (T4) (μg/dL)	10.11 ± 1.20	8.41 ± 1.43^a^	10.05 ± 1.94	7.76 ± 2.01^a^	4.5–14.5
Thyroid‐stimulating hormone (TSH) Triiodate (μIU/mL)	2.68 ± 1.46	2.72 ± 0.97	2.50 ± 1.82	2.74 ± 2.22	0.35–5.5

*Note:* Data is represented as Mean ± SD (percent change). The data was analyzed within a group using the dependent Student's *t*‐test and between groups using the independent Student's *t*‐test. Statistically significant at *p*‐value < 0.05.

^a^ Significant *p*‐value within the group.

### 3.7 Effect of WS Root Extract on Cardiac Health

C‐Reactive Protein (CRP), a protein produced by the liver as a response to inflammation, is one of the biomarkers of cardiac health. Elevated CRP levels are a strong indicator of inflammation in the body. The test group showed a significant decrease in CRP levels (51.1%). However, no statistically significant improvement was seen in the control group. The study findings indicate that WS root extract has anti‐inflammatory properties, as evidenced by its potential to reduce CRP levels. NT‐proBNP (N‐terminal pro‐B‐type natriuretic peptide) is a cardiac biomarker secreted in response to ventricular wall stress and volume overload, and it is widely used for the diagnosis, prognosis, and risk stratification of heart failure. It serves as a sensitive biomarker for cardiac function (Muscari et al. 2021; Raveendran et al. 2025). Its levels also vary with age and sex and have been proposed as a marker of biological aging and mortality risk in older adults (Braisch et al. 2022; Cao et al. 2019). In the test group, there was a decrease in NT‐pro BNP levels (28.70%) compared to a slight increase in the control group. The levels of NT‐pro BNP were in the normal physiological range; the WS root extract afforded further cardiac protection by reducing the marker. The effect of WS root extract and placebo capsules on CRP and NT‐pro BNP is depicted in Table 7.

**TABLE 7:** Effect of WS root extract and placebo capsule on CRP and NT‐pro BNP levels.

Parameters	Test (*n* = 20)		Placebo (*n* = 20)		Reference range
	Screening	Day 180	Screening	Day 180	
CRP (mg/dL)	5.88 ± 3.37	2.88 ± 1.09^a^	4.66 ± 4.30	3.37 ± 1.08	< 6
%Change	51.10%		27.77%		
NT‐pro BNP (pg/mL)	106.32 ± 166.43	75.81 ± 52.28	75.63 ± 78.53	79.14 ± 61.90	50 to 75 years: ≤ 900 (Acute heart failure)
%Change	28.70%		4.63%		

*Note:* Data is represented as Mean ± SD. Analysis was done using the independent Student's *t*‐test and Mann–Whitney *U* test for between groups and the dependent Student's *t*‐test and Wilcoxon signed‐rank test for within groups. Statistically significant at *p*‐value < 0.05.

^a^ Significant *p*‐value within the group.

### 3.8 Effect of WS on Immune Function, Glycemic Profile, and Cortisol Levels

The effect of WS on the immune system was evaluated by analyzing the level of CD3, CD4, and CD8 cells of each participant during screening and upon oral treatment for 180 days. The data demonstrated that the test group showed a slight improvement in CD3, CD4, and CD8 levels, and the placebo showed a significant increase in CD3 and CD8. However, all parameters including CD3 and CD8 remained within reference ranges throughout the study and were clinically non‐significant (*p* > 0.05) in nature.

Glycemic status assessments revealed that fasting blood glucose levels remained normal in the test group but significantly increased in the control group. Fasting insulin levels decreased significantly in the test group, while the control group showed an increase. In the test group, 2 prediabetic and 2 diabetic participants at baseline saw improvements, with 3 normalizing and 1 remaining diabetic post‐study. In the placebo group, 2 prediabetic and 3 diabetic participants progressed, with 9 becoming prediabetic, 2 remaining diabetic, and 1 converting to prediabetic. This data is summarized in Table 8.

**TABLE 8:** Effect of WS root extract capsule on immune function, sugar, and cortisol level.

Parameters	Test (*n* = 20)		Placebo (*n* = 20)		Reference range
	Screening	Day 180	Screening	Day 180	
CD3 (%)	72.89 ± 8.38	75.00 ± 7.15	76.35 ± 7.78	79.80 ± 6.86^a^	60–90
CD4 (%)	41.20 ± 7.34	42.35 ± 6.67	42.61 ± 7.68	42.75 ± 6.38	30–50
CD8 (%)	30.63 ± 5.28	31.60 ± 5.11	31.00 ± 6.97	34.85 ± 7.71^a^	10–35
**Cortisol**					
Cortisol (nmol/L)	96.36 ± 29.60	84.74 ± 28.13	96.49 ± 30.38	93.89 ± 30.68	83–359
%Change	12.05%		2.69%		
**Glycemic status**					
Fasting blood Glucose (mg/dL)	88.78 ± 7.94	91.67 ± 6.85	85.55 ± 8.42	101.78 ± 16.92^a^, ^b^	Normal: 70–99
Fasting insulin (mIU/L)	13.13 ± 8.44	8.63 ± 6.36^a^, ^b^	13.53 ± 5.01	16.57 ± 13.35	0.2–25.0
HbA1C (%)	5.58 ± 0.58	5.56 ± 0.36	5.60 ± 0.81	5.75 ± 0.50	Non‐diabetic adults < 6.0
					Prediabetes = 6.0–6.4
					Diabetes = ≥ 6.5

*Note:* Data is represented as Mean ± SD (percent change). The data was analyzed within a group by using the dependent Student's *t*‐test and independent Student *t*‐test between groups. Statistically significant at *p*‐value < 0.05.

^a^ Significant *p*‐value within the group.

^b^ Significant *p*‐value between groups.

### 3.9 Assessment of Chest X‐Ray and ECG

No clinically significant abnormalities or changes were detected in the ECG and chest X‐ray examinations both at screening and at the end of the study.

### 3.10 Assessment of Vital Signs

No clinically significant changes were observed in blood pressure, pulse rate, respiratory rate, and oral temperature across both groups throughout the study. All vital signs remained within normal limits for all groups. All 40 participants were compliant and showed excellent tolerability to the investigational product.

### 3.11 Standardization and Quality Control of WS Root, Extract, and Capsule

**FIGURE 2:**
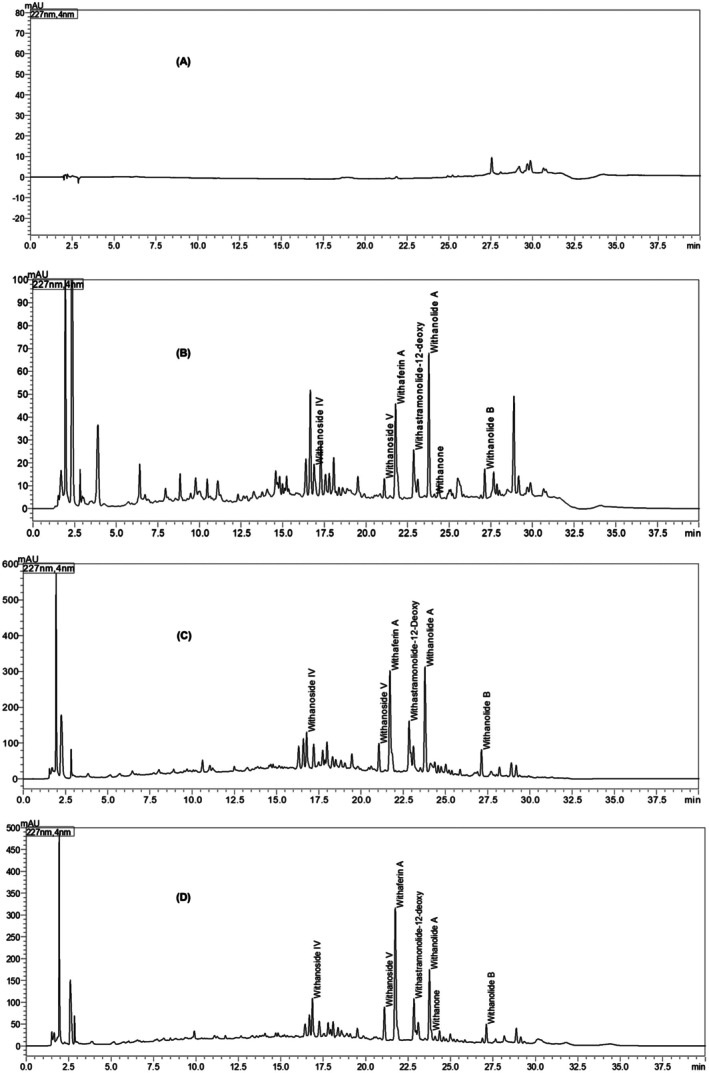
HPLC Chromatogram of (A) Blank Plasma; (B) *Withania somnifera* roots; (C) *Withania somnifera* root extract; (D) *Withania somnifera* root extract capsules.

The WS root, extract, and capsules were analyzed for the content of withanosides and withanolides per the USP‐NF WS root monograph. The total withanolides in the extract were found to be NLT 2.5% w/w whereas each capsule of WS root extract consists of NLT 5.00 mg total withanolides. The quality control parameters of the WS capsule are depicted in Table 9. Standardization of extract and capsules using HPLC‐PDA is represented in Figure 2.

**TABLE 9:** Quality control parameters of WS root extract capsules.

Name of product	*Withania somnifera* (L.) Dunal root extract (2.5%) Capsule (Each capsule contains 200 mg of WS root extract)
Brand Name	LongeFera
Plant part used	Roots
Physical characteristics	Description of capsule
	Yellow/yellow color veggie capsule, size “0”
	Appearance of Extract
	Light to dark brown free‐flowing powder
Chemical analysis	Total Withanolides NLT 5.00 mg/Capsule
Disintegration time	NMT 30 min
Weight variation	500 mg ±7.5%
Impurities	Lead: < 0.50 ppm Mercury: < 0.50 ppm Arsenic: < 1.00 ppm Cadmium: < 0.50 ppm Residual solvents: Meets USP‐NF <467>
Microbial analysis	Total plate count: < 1000 cfu/g Yeast & Mold: < 100 cfu/g Coliform: < 3.0 mpn/g Enterobacterial Count: < 100.00 cfu/g *E.coli*: Absent *Salmonella*: Absent *Staphylococcus aureus* : Absent
Shelf life	24 months from the date of manufacture
Packaging	65 Capsules in white color round HDPE bottles

## 4 Discussion

Over the 180‐day study period, standardized *Withania somnifera* root extract demonstrated a strong safety profile, with no significant adverse events reported. No clinically significant changes were seen in vital signs (temperature, pulse, respiratory rate, blood pressure) or hematological, hepatic, renal, lipid, and thyroid parameters, with all values remaining within clinically normal limits before and after the study, thus reinforcing the extract's safety for long‐term use. Key findings include a significant increase in levels of testosterone in males. A significant reduction, that is, 51.1% in CRP and 28.70% in NT‐pro BNP suggests anti‐inflammatory benefits.

WS exerts anti‐inflammatory effects through withanolides like withaferin‐A, which inhibit TNF‐α, IL‐1β, and NF‐κB pathways, reducing oxidative stress, neutrophil infiltration, and tissue inflammation. Additionally, WS enhances testosterone production by stimulating the hypothalamic–pituitary–gonadal axis, increasing luteinizing hormone secretion and testosterone levels. This action supports its therapeutic potential in reducing inflammation and improving the anabolic hormone to support muscle health (Paul et al. 2021).

The findings from the present study align with previous research demonstrating the tolerability and safety of WS root extract. A study on WS capsules, administered 1000 mg/day to healthy male participants for 4 weeks, reported no AEs and observed no significant changes in safety parameters such as liver, kidney, and thyroid functions. These results enhance enthusiasm for the safety of WS supplementation in healthy populations (Vaidya et al. 2024).

Previous studies, including a randomized, placebo‐controlled, double‐blind trial conducted earlier, supported the safety of WS root extract at 300 mg twice daily in healthy adults. In consonance with this study's findings, this trial observed no significant AEs or abnormalities in vital signs, biochemical, hematological, or thyroid parameters across an 8‐week period, reinforcing the safety profile of WS for regular use (Verma et al. 2021).

The safety findings from the present study are consistent with those reported in a dose‐escalation study that evaluated the tolerability of WS in healthy volunteers at doses ranging from 750 to 1250 mg daily. In that open‐label trial (Raut et al. 2012), the WS formulation was well tolerated, with only one adverse event leading to participant withdrawal. Muscle strengthening, lipid‐lowering effects, and improved sleep quality were also noted. At the same time, all hematological and biochemical organ function tests remained within normal ranges, further supporting the safety profile of WS in escalating doses (Raut et al. 2012).

Smith et al. (2023) have demonstrated WS exceptional influence on hormone regulation, particularly its ability to significantly boost free testosterone in men. Consistent with these findings, the present study observed a marked increase in testosterone levels among male participants following WS root extract supplementation.

The strength of the study lies in its double‐blind, placebo‐controlled design and extended duration, which provide a gestalt assessment of the long‐term effects and/or safety of WS supplementation. However, a limitation of the study is its small sample size, which may restrict the overall generalizability of the key findings to larger, more diverse populations. Another major limitation is that the study provides data only for male participants.

In summary, *Withania somnifera* root extract offers a safe oral nutraceutical, with its potential benefits establishing a significant impact on health outcomes in males. The findings of this study underscore the safety profile of WS supplementation, offering valuable insights that can support evidence‐based decision‐making in health strategies for healthcare professionals and consumers.

## 5 Conclusion

This 180‐day randomized, placebo‐controlled, double‐blind trial provides robust evidence of the safety and tolerability of standardized WS root extract capsules in healthy adult participants. The anthropometric parameters, hematological, hepatic, renal, and lipid profiles, male reproductive hormones, thyroid function, glycemic indices, and cortisol levels were within normal ranges after treatment. Notably, significant reductions were observed in inflammatory markers (CRP), NT‐pro BNP levels, and slight improvements in immunity markers. Furthermore, assessments of vital signs, chest X‐rays, and ECG revealed no abnormalities, and no adverse events were reported. These findings support the safety of standardized WS root extract for long‐term consumption and highlight its potential as a safe nutraceutical intervention to support health and well‐being in human populations.

## Author Contributions

Conceptualization: All authors; Data collection: N.V., R.A., P.K., G.G., D.N.; Data analysis and interpretation: N.V., R.A., P.K., G.G., D.N.; Writing the original draft: S.N.; Writing, review, and editing: S.N.; Supervision: A.M., A.V., S.N.; All the authors have read and approved the final version of the manuscript.

## Funding

This project is funded by Phytoveda Pvt. Ltd., Mumbai, India.

## Conflicts of Interest

LongeFera is globally marketed by Phytoveda Pvt. Ltd., Mumbai, India, which funded this study. A.M., A.V., and S.N. are employees of Phytoveda Pvt. Ltd., Mumbai, India and Viridis Biopharma Pvt. Ltd., Mumbai, India. However, these authors had no role in the trial or analysis of results. The clinical trial was conducted by an external Contract Research Organization (CRO), MPREX Healthcare Pvt. Ltd., Pune, India, who analyzed the results themselves along with the clinicians conducting the study in this multicentric trial (N.V., R.A., P.K., G.G., D.N.).

## Data Availability

The data generated during and/or analyzed during the current clinical study are available from the corresponding author upon request.

## References

[fsn371388-bib-0001] Baker, C. , J. B. Kirby , J. O'Connor , K. G. Lindsay , A. Hutchins , and M. Harris . 2022. “The Perceived Impact of Ashwagandha on Stress, Sleep Quality, Energy, and Mental Clarity for College Students: Qualitative Analysis of a Double‐Blind Randomized Control Trial.” Journal of Medicinal Food 25: 1095–1101. 10.1089/jmf.2022.0042. 35984870

[fsn371388-bib-0002] Basudkar, V. , G. Gujrati , S. Ajgaonkar , M. Gandhi , D. Mehta , and S. Nair . 2024. “Emerging Vistas for the Nutraceutical *Withania somnifera* in Inflammaging.” Pharmaceuticals 17: 597. 10.3390/ph17050597. 38794167 PMC11123800

[fsn371388-bib-0041] Berra, J. L. , S. Chernigoy , K. Alvarez , and M. J. Wernisch . 2024. “Ashwagandha Under Fire: A Critical Scientific Analysis of Regulatory Decisions.” International Journal of Ayurveda Research 5, no. 3: 148–153. 10.4103/ijar.ijar_192_24.

[fsn371388-bib-0004] Biswal, B. M. , S. A. Sulaiman , H. C. Ismail , H. Zakaria , and K. I. Musa . 2013. “Effect of *Withania somnifera* (Ashwagandha) on the Development of Chemotherapy‐Induced Fatigue and Quality of Life in Breast Cancer Patients.” Integrative Cancer Therapies 12: 312–322. 10.1177/1534735412464551. 23142798

[fsn371388-bib-0005] Bonilla, D. A. , Y. Moreno , C. Gho , J. L. Petro , A. Odriozola‐Martínez , and R. B. Kreider . 2021. “Effects of Ashwagandha (*Withania somnifera*) on Physical Performance: Systematic Review and Bayesian Meta‐Analysis.” Journal of Functional Morphology and Kinesiology 6: 20. 10.3390/jfmk6010020. 33670194 PMC8006238

[fsn371388-bib-0006] Braisch, U. , W. Koenig , D. Rothenbacher , et al. 2022. “N‐Terminal Pro Brain Natriuretic Peptide Reference Values in Community‐Dwelling Older Adults.” ESC Heart Failure 9: 1703–1712. 10.1002/ehf2.13834. 35199488 PMC9065825

[fsn371388-bib-0007] Cao, Z. , Y. Jia , and B. Zhu . 2019. “BNP and NT‐proBNP as Diagnostic Biomarkers for Cardiac Dysfunction in Both Clinical and Forensic Medicine.” International Journal of Molecular Sciences 20: 1820. 31013779 10.3390/ijms20081820 PMC6515513

[fsn371388-bib-0008] Choudhary, B. , A. Shetty , and D. G. Langade . 2015. “Efficacy of Ashwagandha ( *Withania somnifera* [L.] Dunal) in Improving Cardiorespiratory Endurance in Healthy Athletic Adults.” Ayu 36: 63–68. 10.4103/0974-8520.169002. 26730141 PMC4687242

[fsn371388-bib-0009] Dadge, S. D. , N. Tiwari , A. Husain , et al. 2023. “Simultaneous Estimation of Five Biomarkers of Neuroprotective Herb Ashwagandha NMITLI‐118R AF1 in Rat Plasma and Brain Using LC‐ESI‐MS/MS: Application to Its Pharmacokinetic and Stability Studies.” Journal of Chromatography B 1228: 123834. 10.1016/j.jchromb.2023.123834. 37481788

[fsn371388-bib-0010] Deshpande, A. , N. Irani , R. Balkrishnan , and I. R. Benny . 2020. “A Randomized, Double Blind, Placebo Controlled Study to Evaluate the Effects of Ashwagandha ( *Withania somnifera* ) Extract on Sleep Quality in Healthy Adults.” Sleep Medicine 72: 28–36. 10.1016/j.sleep.2020.03.012. 32540634

[fsn371388-bib-0011] Girme, A. , G. Saste , S. Pawar , et al. 2020. “Investigating 11 Withanosides and Withanolides by UHPLC–PDA and Mass Fragmentation Studies From Ashwagandha ( *Withania somnifera* ).” ACS Omega 5: 27933–27943. 10.1021/acsomega.0c03266. 33163776 PMC7643146

[fsn371388-bib-0012] Gopukumar, K. , S. Thanawala , V. Somepalli , T. S. S. Rao , V. B. Thamatam , and S. Chauhan . 2021. “Efficacy and Safety of Ashwagandha Root Extract on Cognitive Functions in Healthy, Stressed Adults: A Randomized, Double‐Blind, Placebo‐Controlled Study.” Evidence‐Based Complementary and Alternative Medicine 2021: 8254344. 10.1155/2021/8254344. 34858513 PMC8632422

[fsn371388-bib-0013] Indian Ministry of AYUSH. Government of India . 2021. Advisory for Refrain From Use of Aswagandha ( *Withania somnjfera* ) Leaves. Goverment of India.

[fsn371388-bib-0014] Joshi, V. , and R. P. Joshi . 2013. “Some Plants Used in Ayurvedic and Homoeopathic Medicine.” Journal of Pharmacognosy and Phytochemistry 2: 269–275.

[fsn371388-bib-0015] Joshi, V. K. , and A. Joshi . 2021. “Rational Use of *Ashwagandha* in *Ayurveda* (Traditional Indian Medicine) for Health and Healing.” Journal of Ethnopharmacology 276: 114101. 10.1016/j.jep.2021.114101. 33831467

[fsn371388-bib-0016] Langade, D. , S. Kanchi , J. Salve , K. Debnath , and D. Ambegaokar . 2019. “Efficacy and Safety of Ashwagandha (*Withania somnifera*) Root Extract in Insomnia and Anxiety: A Double‐Blind, Randomized, Placebo‐Controlled Study.” Cureus 11: e5797. 10.7759/cureus.5797. 31728244 PMC6827862

[fsn371388-bib-0017] Machín, R. P. , M. Florido , R. Chirino‐Godoy , and L. López‐Rios . 2023. “Adaptogenic Botanicals With Emphasis on Rhodiola Rosea and *Withania somnifera* .” European Journal of Medicinal Plants 34: 20–39. 10.9734/ejmp/2023/v34i111168.

[fsn371388-bib-0018] Mahdi, A. A. , K. K. Shukla , M. K. Ahmad , et al. 2011. “ *Withania somnifera* Improves Semen Quality in Stress‐Related Male Fertility.” Evidence‐Based Complementary and Alternative Medicine 2011: 576962. 10.1093/ecam/nep138. 19789214 PMC3136684

[fsn371388-bib-0019] Malik, F. , A. Kumar , S. Bhushan , et al. 2009. “Immune Modulation and Apoptosis Induction: Two Sides of Antitumoural Activity of a Standardised Herbal Formulation of *Withania somnifera* .” European Journal of Cancer 45: 1494–1509. 10.1016/j.ejca.2009.01.034. 19269163

[fsn371388-bib-0020] Meher, S. K. , B. Das , P. Panda , G. C. Bhuyan , and M. M. Rao . 2016. “Uses of *Withania somnifera* (Linn) Dunal (Ashwagandha) in Ayurveda and Its Pharmacological Evidences.” Research Journal of Pharmacology and Pharmacodynamics 8: 23–29. 10.5958/2321-5836.2016.00006.9.

[fsn371388-bib-0021] Ministry of Ayush, Government of India, New Delhi . 2024. “Ashwagandha (*Withania somnifera*) Safety Dossier 2.0.” Central Council for Research in Ayuredic Sciences.

[fsn371388-bib-0022] Misico, R. I. , V. E. Nicotra , J. C. Oberti , G. Barboza , R. R. Gil , and G. Burton . 2011. “Withanolides and Related Steroids.” Progress in the Chemistry of Organic Natural Products 94: 127–229. 10.1007/978-3-7091-0748-5_3. 21833839

[fsn371388-bib-0023] Modi, S. J. , A. Tiwari , C. Ghule , et al. 2022. “Pharmacokinetic Study of Withanosides and Withanolides From *Withania somnifera* Using Ultra‐High Performance Liquid Chromatography‐Tandem Mass Spectrometry (UHPLC‐MS/MS).” Molecules 27: 1476. 10.3390/molecules27051476. 35268576 PMC8912008

[fsn371388-bib-0024] Mohanty, I. , D. S. Arya , A. Dinda , K. K. Talwar , S. Joshi , and S. K. Gupta . 2004. “Mechanisms of Cardioprotective Effect of *Withania somnifera* in Experimentally Induced Myocardial Infarction.” Basic & Clinical Pharmacology & Toxicology 94: 184–190. 10.1111/j.1742-7843.2004.pto940405.x. 15078343

[fsn371388-bib-0025] Muscari, A. , G. Bianchi , P. Forti , et al. 2021. “N‐Terminal Pro B‐Type Natriuretic Peptide (NT‐proBNP): A Possible Surrogate of Biological Age in the Elderly People.” GeroScience 43: 845–857. 10.1007/s11357-020-00249-2. 32780292 PMC8110633

[fsn371388-bib-0026] Pandit, S. , A. K. Srivastav , T. K. Sur , S. Chaudhuri , Y. Wang , and T. K. Biswas . 2024. “Effects of *Withania somnifera* Extract in Chronically Stressed Adults: A Randomized Controlled Trial.” Nutrients 16: 1293. 10.3390/nu16091293. 38732539 PMC11085552

[fsn371388-bib-0027] Patwardhan, B. , S. Chaturvedi , G. Tillu , S. Deshpande , and B. M. Hegde . 2024. “Danish Ban on Ashwagandha: Truth, Evidence, Ethics, and Regulations.” Journal of Ayurveda and Integrative Medicine 15: 101028. 10.1016/j.jaim.2024.101028. 38969606 PMC11403136

[fsn371388-bib-0028] Paul, S. , S. Chakraborty , U. Anand , et al. 2021. “ *Withania somnifera* (L.) Dunal (Ashwagandha): A Comprehensive Review on Ethnopharmacology, Pharmacotherapeutics, Biomedicinal and Toxicological Aspects.” Biomedicine & Pharmacotherapy 143: 112175. 10.1016/j.biopha.2021.112175. 34649336

[fsn371388-bib-0029] Raut, A. A. , N. N. Rege , F. M. Tadvi , et al. 2012. “Exploratory Study to Evaluate Tolerability, Safety, and Activity of Ashwagandha (*Withania somnifera*) in Healthy Volunteers.” Journal of Ayurveda and Integrative Medicine 3: 111–114. 10.4103/0975-9476.100168. 23125505 PMC3487234

[fsn371388-bib-0030] Raveendran, H. , A. Sukumaran , A. Krishnan , J. Paul , and D. Vasudevan . 2025. “N‐Terminal Pro‐Brain Natriuretic Peptide; Current Trends in Diagnostics.” IP Indian Journal of Immunology and Respiratory Medicine 9: 3–8. 10.18231/j.ijirm.2024.002.

[fsn371388-bib-0039] Ruknuddin, G. , G. Tillu , A. Ahmad , and R. Kaushik . 2023. “Ashwagandha (*Withania somnifera*) Safety Dossier.” Indian Ministry of AYUSH, 81.

[fsn371388-bib-0032] Saha, P. , S. Ajgaonkar , D. Maniar , S. Sahare , D. Mehta , and S. Nair . 2024. “Current Insights Into Transcriptional Role(s) for the Nutraceutical *Withania somnifera* in Inflammation and Aging.” Frontiers in Nutrition 11: 1370951. 10.3389/fnut.2024.1370951. 38765810 PMC11099240

[fsn371388-bib-0033] Saleem, S. , G. Muhammad , M. A. Hussain , M. Altaf , and S. N. A. Bukhari . 2020. “ *Withania somnifera* L.: Insights Into the Phytochemical Profile, Therapeutic Potential, Clinical Trials, and Future Prospective.” Iranian Journal of Basic Medical Sciences 23: 1501–1526. 10.22038/IJBMS.2020.44254.10378. 33489024 PMC7811807

[fsn371388-bib-0042] Sharma, E. , G. Ganu , K. Kshirsagar , et al. 2025. “An Open‐Label, Single Dose, Safety and Pharmacokinetic Study of *Withania somnifera* Root Extract in Healthy Volunteers.” Drug Metabolism and Personalized Therapy 40, no. 1: 23–34. 10.1515/dmpt-2024-0089. 39963957

[fsn371388-bib-0034] Shinde, S. , A. K. Balasubramaniam , V. Mulay , G. Saste , A. Girme , and L. Hingorani . 2023. “Recent Advancements in Extraction Techniques of Ashwagandha ( *Withania somnifera* ) With Insights on Phytochemicals, Structural Significance, Pharmacology, and Current Trends in Food Applications.” ACS Omega 8: 40982–41003. 10.1021/acsomega.3c03491. 37970011 PMC10633886

[fsn371388-bib-0035] Smith, S. J. , A. L. Lopresti , and T. J. Fairchild . 2023. “Exploring the Efficacy and Safety of a Novel Standardized Ashwagandha (*Withania somnifera*) Root Extract (*Witholytin*) in Adults Experiencing High Stress and Fatigue in a Randomized, Double‐Blind, Placebo‐Controlled Trial.” Journal of Psychopharmacology 37: 1091–1104. 10.1177/02698811231200023. 37740662 PMC10647917

[fsn371388-bib-0036] Vaidya, V. G. , A. Gothwad , G. Ganu , A. Girme , S. J. Modi , and L. Hingorani . 2024. “Clinical Safety and Tolerability Evaluation of *Withania somnifera* (L.) Dunal (Ashwagandha) Root Extract in Healthy Human Volunteers.” Journal of Ayurveda and Integrative Medicine 15: 100859. 10.1016/j.jaim.2023.100859. 38154316 PMC10784694

[fsn371388-bib-0037] Verma, N. , S. K. Gupta , S. Tiwari , and A. K. Mishra . 2021. “Safety of Ashwagandha Root Extract: A Randomized, Placebo‐Controlled, Study in Healthy Volunteers.” Complementary Therapies in Medicine 57: 102642. 10.1016/j.ctim.2020.102642. 33338583

[fsn371388-bib-0038] Wijeratne, E. M. K. , Y.‐M. Xu , R. Scherz‐Shouval , et al. 2014. “Structure–Activity Relationships for Withanolides as Inducers of the Cellular Heat‐Shock Response.” Journal of Medicinal Chemistry 57: 2851–2863. 10.1021/jm401279n. 24625088

